# Induction of immunogenic necroptosis by shikonin in drug-resistant head and neck squamous cell carcinoma cells

**DOI:** 10.3892/or.2025.8996

**Published:** 2025-09-24

**Authors:** Satsuki Kishita, Naoki Umemura, Hiromi Miyazaki, Makoto Adachi, Hideki Yagi, Emika Ohkoshi

**Affiliations:** 1 Division of Immunobiology, Graduate School of Pharmaceutical Sciences, International University of Health and Welfare, Ohtawara, Tochigi 324-8501, Japan; 2 Department of Oral Biochemistry, Asahi University School of Dentistry, Mizuho, Gifu 501-0296, Japan; 3 Division of Biomedical Engineering, National Defense Medical College Research Institute, Saitama, Saitama 359-8513, Japan; 4 Department Oral and Maxillofacial Surgery, Nagoya Tokushukai General Hospital, Kasugai, Aichi 487-0016, Japan; 5 Division of Pharmacognosy, Graduate School of Pharmaceutical Sciences, International University of Health and Welfare, Ohtawara, Tochigi 324-8501, Japan

**Keywords:** multidrug resistance, head and neck squamous cell carcinoma, naphthoquinones, necroptosis, reactive oxygen species, oxidative stress, immunotherapy

## Abstract

In recent years, immune checkpoint inhibitors such as nivolumab have been used to treat recurrent or metastatic head and neck cancer. However, some patients do not respond to nivolumab, and the treatment options for these patients are limited. Therefore, identifying compounds for developing new therapeutic strategies for intractable cancer is important. The acquired multidrug-resistant metastatic head and neck squamous cell carcinoma cell line, R HSC-3, expresses refractory cancer-specific proteins such as the drug excretion transporter, ATP binding cassette subfamily G member 2, the cancer stem cell markers, CD44, SRY-box transcription factor 9 and Notch, and the poor prognosis factor, fibroblast growth factor 9, and is a useful *in vitro* model for acquired multidrug resistance. In the present study, compounds that may be more effective than conventional chemotherapeutic drugs in R HSC-3 cells were searched and the cell death mechanism was investigated. The results showed that naphthoquinones inhibited the viability of R HSC-3 cells at low concentrations and induced necroptotic cell death. Naphthoquinone-induced necroptotic cell death in R HSC-3 cells induced the expression of calreticulin, an immunogenic marker. It was further found that mitochondrial-derived reactive oxygen species mediated the oxidative stress damage by naphthoquinone-induced necroptotic cell death in these cells. Moreover, necroptotic cell death by shikonin, a naphthoquinone, may generate immunogenic signals from multidrug-resistant cancer cells. The present study revealed that naphthoquinones may not only induce necroptosis in refractory head and neck cancer cells but also induce tumor immunity. Therefore, naphthoquinones may represent a new avenue for the development of new therapeutic agents targeting multidrug-resistant head and neck cancer.

## Introduction

Head and neck squamous cell carcinoma is a highly malignant cancer with a 5-year survival rate of 30–50% and is the sixth most common cancer worldwide (1). Surgical excision of this tumor type is difficult as it occurs in the complex site of the head and neck. Moreover, even if the tumor is excised, the quality of life of the patient is significantly reduced. Even after reduction or resection of head and neck cancer by chemotherapy or surgery, recurrence or metastasis occurs in 30% of cases (2,3). The average survival time of metastatic/recurrent cases of head and neck squamous cell carcinoma treated with chemotherapy alone is 6–9 months, and the 1-year survival rate is as low as 20–40%. Compared with no treatment, chemotherapy achieves only a slight survival benefit (4).

Drug resistance is a critical obstacle to the effective treatment of patients with cancer. Several multidrug resistance mechanisms have been identified, such as the expression of drug efflux ATP binding cassette (ABC) transporters (such as ABCB1 and ABCG2) that discharge anticancer drugs outside of cancer cells, apoptosis resistance, decreased drug binding as a result of gene mutations and the activation of survival signals (5). To the best of our knowledge, a compound that effectively induces cell death in multidrug-resistant head and neck cancer cells has not been identified.

We previously established a multi-drug resistant cell line, which we named R HSC-3 (6). R HSC-3 cells were generated by long-term repeated treatment of the HCS-3 human metastatic oral squamous cell carcinoma cell line with cisplatin, the first-line treatment drug for oral squamous cell carcinoma. The cells showed a maximum 155-fold higher resistance against anticancer drugs such as SN-38 and docetaxel, which have different mechanisms of action from cisplatin (7). This cell line expresses refractory cancer-specific proteins such as the drug excretion transporter, ABCG2, the cancer stem cell markers, CD44, SRY-box transcription factor 9 (SOX9) and Notch, and the poor prognosis factor, fibroblast growth factor 9 (FGF9) (6). This cell line is a useful *in vitro* model for multidrug resistance.

The present study investigated compounds that effectively induce cell death in multidrug-resistant head and neck cancer cells and explored the mechanism of cell death induction. These findings may lead to the establishment of a new treatment strategy for intractable head and neck cancer with acquired drug resistance.

## Materials and methods

### Reagents

Dulbecco's Modified Eagle's Medium (DMEM) and glucose-free DMEM was obtained from Invitrogen (Thermo Fisher Scientific, Inc.). Fetal bovine serum (FBS; 10%), 1% antibiotics/antimycotic solution 100X (10,000 U/ml penicillin G, 10,000 µg/ml streptomycin, 25 µg/ml amphotericin B) were purchased from HyClone (Cytiva). The anti-receptor interacting serine/threonine kinase 1 (RIP1/RIPK1) mouse monoclonal antibody was purchased from BD Biosciences (cat. no. 610458). Anti-calreticulin rabbit monoclonal antibody (cat. no. C4606) and anti-β-actin mouse monoclonal antibody (cat. no. A5441) were obtained from Sigma-Aldrich (Merck KGaA). Anti-gasdermin D rabbit monoclonal antibody (cat. no. 97558) and horseradish peroxidase (HRP)-labeled goat anti-rabbit IgG (cat. no. 7074) was from Cell Signaling Technology, Inc. Goat anti-mouse IgG-HRP antibody was from Santa Cruz Biotechnology, Inc. (cat. no. sc-2005) and rabbit anti-mouse IgG H&L (HRP) was purchased from Abcam (cat. no. ab6728). The RIP1 inhibitor, 5-((7-chloro-1*H*-indol-3-yl)methyl)-3-methylimidazolidine-2,4-dione (7-Cl-O-Nec1) was obtained from Merck KGaA and the mixed lineage kinase domain-like protein (MLKL) inhibitor, necrosulfonamide (NSA) was from R&D Systems, Inc. *N*-acetyl cysteine (NAC) and 2-deoxy-D-glucose (2DG) were purchased from FUJIFILM Wako Pure Chemical. Shikonin, acetyl shikonin and β-hydroxyisovaleryl shikonin were purchased from Nagara Science Co., Ltd. The Pierce BCA Protein Assay Kit was purchased from Thermo Fisher Scientific, Inc. and the Cell Proliferation Kit I (MTT) was obtained from Roche Diagnostics. The JC-1 MitoMP Detection Kit was purchased from Dojindo Laboratories, Inc. The CellTiter-Glo^®^ Luminescent Cell Viability Assay and ROS-Glo™ Assay Kit were obtained from Promega Corporation.

### Cell culture

We established the R HSC-3 multi-drug resistant oral squamous cell carcinoma cell line in a previous study (6). R HSC-3 cells were generated by long-term repeated treatment of the HSC-3 human metastatic oral squamous cell carcinoma cell line (Riken Cell Bank; cat. no. RBC1975) with cisplatin. The R HSC-3 line was cultured in DMEM with inactivated 10% FBS and 1% antibiotic/antimycotic solution 100X at 37°C with 5% CO_2_.

### Cell viability assays

Two types of cell viability assays were used: The MTT assay and the ATP-based CellTiter-Glo^®^ Luminescent Cell Viability Assay, which assess mitochondrial activity and cellular energy status, respectively. Using both assays provided complementary and comprehensive assessment of cytotoxicity. R HSC-3 cells were plated in a 96-well plate (TPP Techno Plastic Products AG) at 1×10^5^ cells/ml and incubated for 24 h. Naphthoquinones were then added at various concentrations (shikonin 1–20 µM, acetyl shikonin 1–30 µM and β-hydroxyisovaleryl shikonin 1–50 µM) for an additional 48 h. For the MTT assay, MTT [3-(4,5-dimethylthiazol-2-yl)-2,5-diphenyltetrazolium bromide; Cell Proliferation Kit I] was added, and the insoluble formazan product was dissolved with 10% SDS in 0.01 M HCl. Absorbance was measured at a wavelength of 595 nm using TECAN Infinite M Plex (Tecan Group, Ltd.). For the ATP assay, the ATP extraction reagent (CellTiter-Glo^®^ Luminescent Cell Viability Assay) was added according to the manufacturers' instructions. The plates were incubated for 1 h, then shaken at room temperature for 1 h and luminescence was measured in relative light units at wavelengths of 485/520 nm using TECAN Infinite M Plex, multimode microplate reader (Tecan Group, Ltd.). In some experiments, cells were pre-treated with 5 mM NAC for 24 h, and in some experiments, 2.5 mM NAC was co-administered with the naphthoquinones.

### Measurement of reactive oxygen species (ROS)

R HSC-3 cells were plated in a 96-well plate at 1×10^5^ cells/ml and incubated for 24 h. The medium was changed from DMEM with 10% FBS to glucose-free DMEM with 10% FBS and 10 nM 2DG (2DG-DMEM). After 24 h of incubation, 20 µM shikonin or 30 µM acetyl shikonin was added to the media. After 6 h of treatment, ROS was measured using the ROS-Glo™ Assay following the manufacturer's protocol. In some experiments, 50 µM 7-Cl-O-Nec1, a RIP1 inhibitor, and 10 µM NSA, a MLKL inhibitor, were first incubated with the cells for 24 h. Naphthoquinones were then administered to the pretreated cells and the ROS levels were measured using the ROS-Glo™ Assay.

### Measurement of mitochondrial toxicity

The Mitochondrial ToxGlo™ Assay (Promega Corporation) was used to measure mitochondrial toxicity. Cell membrane integrity was first assessed by measuring the presence or absence of a distinct protease activity associated with necrosis using a fluorogenic peptide substrate (bis-alanyl-alanyl-phenylalanyl-rhodamine 110; bis-AAF-R110) to measure the dead cell marker protease activity. Next ATP was measured by adding the ATP extraction reagent, resulting in cell lysis and the generation of a luminescent signal proportional to the level of ATP present. R HSC-3 cells were plated in a 96-well plate at 1×10^5^ cells/ml and cultured in glucose-free DMEM supplemented with 1 µM galactose and 10% FBS at 37°C for 24 h. Various concentrations of naphthoquinones were added and the cells were cultured for 24 h. Next, 5X Cytotoxicity Reagent (Mitochondrial ToxGlo™ Assay) was added and the cells were shaken at room temperature for 1 min, then incubated at 37°C for 30 min. Fluorescence was then measured at wavelengths of 485/520 nm using a multimode microplate reader. In some experiments, the cells were pretreated with 2.5 mM NAC for 24 h or 1.25 mM NAC was co-administered with the naphthoquinones. Oligomycine (FUJIFILM Wako Pure Chemical Corporation) was used as a positive control inhibitor of oxidative phosphorylation (OXPHOS).

### Lentiviral transduction and fluorescent live cell imaging

Calreticulin-red fluorescent protein (RFP) cell lines were generated as follows. R HSC-3 cells were plated in a 6-well plate at 1×10^5^ cells/ml in DMEM with 10% FBS and incubated at 37°C for 24 h. R HSC-3 cells were transduced with the pre-packaged LentiBrite™ Calreticulin-RFP-KDEL Lentiviral Biosensor (Merck KGaA; cat. no. 17-10146) at a multiplicity of infection of 20 and incubated at 37°C with 5% CO_2_ for 24 h. Lentivirus-containing medium was removed and replaced with fresh growth medium. Cells were cultured for another 24–48 h, with medium changes every 24 h. RFP expression was observed with a fluorescence microscope (Olympus IX70; Olympus Corporation).

Lentivirus-infected R HSC-3 cells were seeded at 1×10^5^ cells/ml in a 96-well microplate and cultured in DMEM with 10% FBS at 37°C for 24 h. Hoechst 33342 was added and the cells were incubated at 37°C for 20 min. Naphthoquinones were then added, and images were obtained and evaluated using an HS all-in-one fluorescence microscope (BIOREVO BZ-9000; Keyence Corporation) and data processing software (BZ-II observation application, BZ-II image analysis application; version 1.42; Keyence Corporation).

### Immunoblot analyses

R HSC-3 cells were treated with 1, 3, 5, 6 or 8 µM shikonin in 2DG-DMEM for 5 h. Whole cell extracts were obtained using 10X RIPA buffer (Cell Signaling Technology, Inc.) with 1 mM PMSF plus protease inhibitor cocktail (Complete, EDTA-free; Roche Diagnostics). Total protein concentration in the lysates was assayed using the Pierce^TM^ BCA Protein Assay Kit (Thermo Fisher Scientific, Inc.). Protein extracts (50 µg) were separated on 8% SDS-PAGE gels and transferred to a polyvinylidene difluoride membrane. Membranes were blocked with 10% non-fat milk for 1 h at room temperature and then incubated with the following primary antibodies at 4°C overnight: Anti-calreticulin (1:10,000), anti-RIP1 (1:1,000), anti-gasdermin D (1:1,000) and anti-β-actin (1:10,000). Following the primary antibody incubation, the membranes were incubated with secondary HRP-conjugated goat anti-mouse (1:10,000) or anti-rabbit IgG (1:10,000) antibodies for 1 h at room temperature. The bands were visualized using Clarity Western ECL Substrate Peroxide solution (Bio-Rad Laboratories, Inc.). The blots were developed and images analyzed with Luminescent Image Analyzer LAS-3000 (FUJIFILM Corporation) and Science Lab 2001 Image Gauge version 4.0 (FUJIFILM Corporation).

### JC-1 mitochondrial membrane potential assay

Mitochondrial membrane potential (Dj m) was assessed using the JC-1 MitoMP Detection Kit (Dojindo Laboratories, Inc.) according to the manufacturer's instructions. Cells were seeded and treated with naphthoquinones as aforementioned. Cells were incubated with 1 mM JC-1 dye at 37°C for 30 min, followed by Hoechst 33342 staining (1:2,000 in PBS) for 1 h. Fluorescence images were acquired using a BZ-X810 all-in-one fluorescence microscope (Keyence Corporation) and analyzed with BZ-H4CM software (Keyence Corporation).

### Statistical analysis

All quantitative data are presented as mean ± SD from at least three independent experiments. The unpaired Student's t-test was used to analyze statistical differences between two groups of data, while two-way ANOVA followed by Tukey's post hoc test was applied for multiple comparisons. P<0.05 was considered to indicate a statistically significant difference. Statistical analysis was performed using Mac Ver.3.0 (Esumi Co. Ltd.) and EZR software (Easy R) version 1.68 (Saitama Medical Center, Jichi Medical University) (8).

## Results

### Naphthoquinones induce cell death of multidrug-resistant oral squamous cell carcinoma cells

We previously generated a multidrug-resistant head and neck squamous cell line, named R HSC-3, by culturing the HSC-3 human head and neck squamous cell carcinoma cell line in cisplatin-containing media (6). R HSC-3 cells were 29 times more resistant to cisplatin than wild-type HSC-3 cells. In the present study the half maximal inhibitory concentration (IC_50_) values of various anticancer drugs and naphthoquinones in R HSC-3 cells were examined using MTT assays. The IC_50_ values of all examined drugs were higher in R HSC-3 cells compared with parental cells (Table I). Notably, the IC_50_ values of shikonin and acetylshikonin, which are naphthoquinones, were ≤3-fold higher in R HSC-3 cells compared with parental cells and did not show a significant difference between the parental strain and resistant strain. These data indicated that naphthoquinones may exhibit potency against multidrug-resistant cancer cells.

### Naphthoquinones induce expression of the necroptosis-associated protein, RIP1

**Figure 1.**
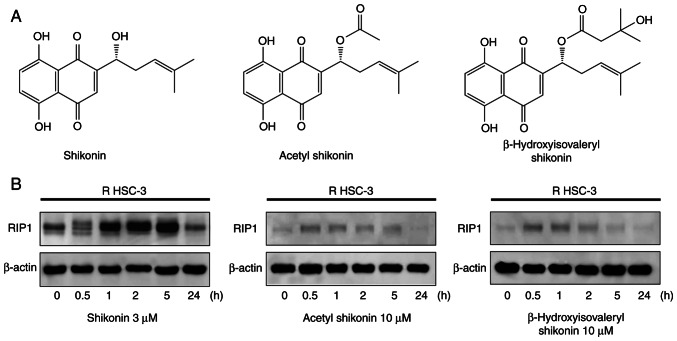
Induction of RIP1 by naphthoquinones in drug-resistant head and neck squamous cell carcinoma cells. (A) Chemical structures of naphthoquinones. (B) Western blot analysis of RIP1 in R HSC-3 cells treated with naphthoquinones for the indicated times. RIP1, receptor interacting protein 1 kinase.

Naphthoquinones are natural red pigment components isolated from the root of *Lithospermum erythrorhizon*. The chemical structures of 1,4-naphthoquinones, including shikonin (5,8-dihydroxy-2-[(1R)-1-hydroxy-4-methyl-3-penten-1-yl]-1,4-naphthalenedione), acetylshikonin and β-hydroxyisovaleryl shikonin, are shown in Fig. 1A. Shikonin is a compound with an OH group at the β-position of the isoprene side chain of the naphthoquinone skeleton. Acetyl shikonin and β-hydroxyisovaleryl shikonin have side chains in which an acetyl group and a β-hydroxyisovaleryl group, respectively, are ester-bonded as substituents to the OH group. The mechanism of cell death of R HSC-3 cells caused by naphthoquinones were next examined. The induction of cleaved poly(ADP-ribose) polymerase (PARP), a marker of apoptosis induction, was observed by immunoblot, but reproducible cleaved PARP expression could not be obtained (data not shown). Therefore, other forms of cell death were explored. RIP1 is a kinase involved in necroptosis, one of the forms of non-apoptotic cell death. Treatment with all naphthoquinones led to the induction and subsequent decrease of RIP1 within 0.5 to 5 h (Fig. 1B). The observed decrease in RIP1 expression after its initial induction may reflect its degradation through caspase-dependent or proteasomal pathways, which has been previously reported (9).

### Effect of naphthoquinones on cell viability and the involvement of oxidative stress

**Figure 2.**
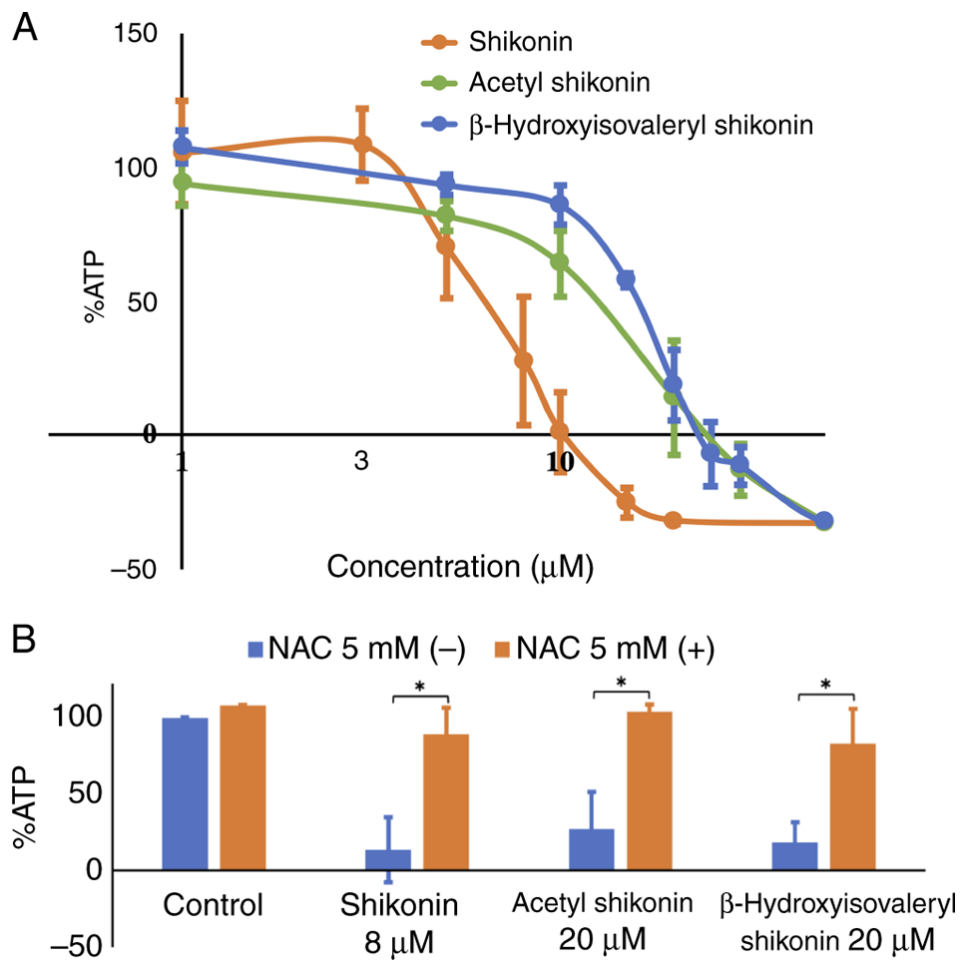
Effect of oxidative stress on naphthoquinone-induced cell death. (A) The effect of naphthoquinones on the viability of R HSC-3 cells. (B) The effect of NAC pretreatment on the cell viability of naphthoquinone-treated R HSC-3 cells. *P<0.05. Data are presented as the mean ± SD (n≥3). NAC, *N*-acetyl cysteine.

Next, the effects of naphthoquinones on the cell viability of drug-resistant cells were evaluated using assays that quantify the ATP levels. The treatment of R HSC-3 cells with all naphthoquinones reduced the ATP levels in a concentration-dependent manner, reflecting cell death (Fig. 2A). The IC_50_ values of shikonin, acetyl shikonin and β-hydroxyisovaleryl shikonin were 6.0, 12.7 and 15.7 µM, respectively. These results indicate that the IC_50_ values of acetyl shikonin and β-hydroxyisovaleryl shikonin, whose OH group is modified, were 2–2.6 times higher than that for shikonin, which has a free OH group on its side chain.

Oxidative stress is a significant inducer of necroptosis, primarily through the production of ROS that activate necroptosis signaling pathways (10). Thus, whether the naphthoquinone-induced cell death is related to oxidative stress was next examined using the antioxidant, NAC. Treatment with NAC prevented the cytotoxicity induced by naphthoquinones and restored viability to near control levels (Fig. 2B). Simultaneous treatment of 5 mM NAC significantly increased survival from 27.4 to 103.8% with shikonin at 8 µM (P=0.018), 13.7 to 89.3% with acetyl shikonin at 20 µM (P=0.037) and 18.6 to 83.1% with β-hydroxyisovaleryl shikonin at 20 µM (P=0.025). NAC alone did not cause cytotoxicity. This result indicated that oxidative stress is involved in the induction of cell death in multidrug-resistant head and neck cancer cells caused by naphthoquinones.

### Changes in the mitochondrial membrane potential by naphthoquinones

**Figure 3.**
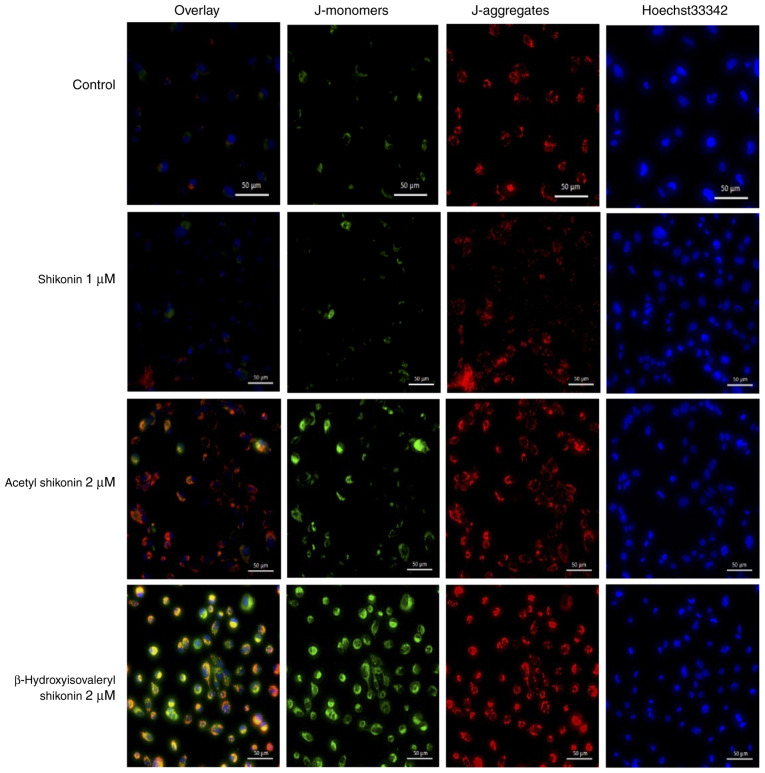
Evaluation of the mitochondrial membrane potential by JC-1 staining of naphthoquinone-treated R HSC-3 cells. R HSC-3 cells treated as indicated were stained with JC-1 (red and green) and Hoechst 33342 (blue). Hoechst33342 was used to stain nuclei and facilitate visualization of all cells. JC-1 staining pattern indicates loss of the mitochondrial membrane potential in treated R HSC-3 cells. Scale bar, 50 µm.

In total, >90% of ROS in cells is produced in mitochondria (11). Excess ROS causes oxidative stress damage in cells and mitochondrial dysfunction (12,13). To examine whether mitochondrial ROS production is involved in oxidative stress-induced cell death caused by naphthoquinones, changes in the mitochondrial membrane potential were investigated using the JC-1 reagent. When the mitochondrial membrane potential is normal, JC-1 emits red fluorescence, and when the membrane potential decreases from apoptosis or necroptosis, JC-1 emits green fluorescence. Hoechst33342 was used to stain nuclei and facilitate visualization of all cells. Treatment of cells with acetyl shikonin and β-hydroxyisovaleryl shikonin for 2 h resulted in mitochondrial membrane potential changes and accumulation of JC-1 monomers, and an increase in red fluorescence and green fluorescence was observed (Fig. 3). This suggests that the oxidative stress that induces cell death in multidrug-resistant head and neck cancer cells by acetyl shikonin and β-hydroxyisovaleryl shikonin is related to the mitochondrial electron transport system. Shikonin did not appear to elicit changes in the mitochondrial membrane potential.

### Effects of changes in intracellular energy metabolism on naphthoquinone-induced cell death

**Figure 4.**
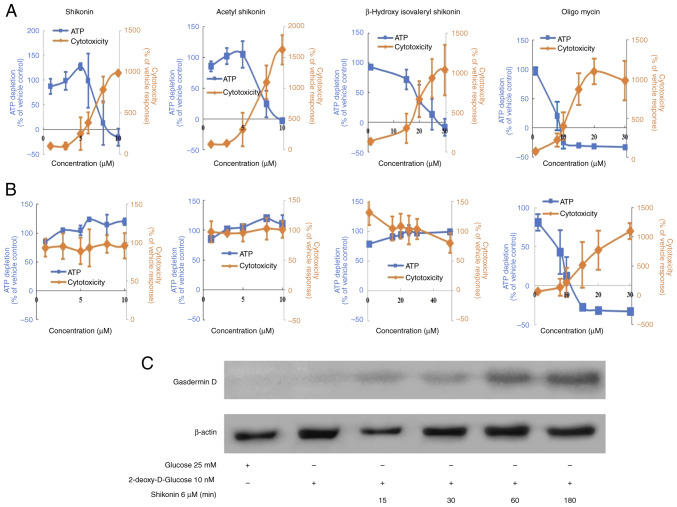
Induction of cell death by naphthoquinones under oxidative phosphorylation-dominant culture conditions. (A) Naphthoquinones induced mitochondria dysfunction in R HSC-3 cells under galactose-containing oxidative conditions. (B) Effects of NAC on naphthoquinone-treated R HSC-3 cells were examined. (C) Expression of gasdermin D in R HSC-3 cells treated with shikonin. Data are presented as the mean ± SD (n≥3). NAC, *N*-acetyl cysteine.

The cell death induced by acetyl shikonin and β-hydroxyisovaleryl shikonin involves the production of ROS but shikonin had no effects on the mitochondrial membrane potential (Fig. 3). One possibility for the difference in effects is that cancer cells, even under aerobic conditions, exhibit enhanced ATP production by the glycolytic pathway rather than mitochondrial OXPHOS (also known as the Warburg effect) (14). ATP production in glycolysis is reported to be ~100 times faster than that in mitochondria (15). Thus, we speculated that it may be difficult to observe mitochondrial membrane changes induced by shikonin in drug-resistant head and neck squamous cells. Therefore, the effect of naphthoquinones on the mitochondrial respiratory chain of R HSC-3 cells were next examined. By switching the carbon source of the medium to galactose, the mitochondrial OXPHOS-dominant state is moderated, and the effects of mitochondrial toxicity and ATP production can be examined (16). To confirm that mitochondrial toxicity is derived from ROS, the antioxidant, NAC, was used. Mitochondrial toxicity was evaluated by simultaneously measuring cytotoxicity (mitochondrial-derived dead cell protease) and ATP production. Cell membrane integrity was first assessed by measuring the presence or absence of a distinct protease activity associated with necrosis using a fluorogenic peptide substrate (bis-AAF-R110) to measure the dead cell marker protease activity. Fluorescence associated with cytotoxicity was measured, ATP extraction reagent was added and luminescence associated with the amount of ATP as a marker of viable cell number was measured (17). As a positive control, oligomycin, which is an OXPHOS inhibitor (complex V inhibitor), was used. Under conditions in which the carbon source in the medium was galactose, the ATP half maximal effective concentration (EC_50_) values for shikonin, acetylshikonin and β-hydroxyisovaleryl shikonin were 7.2, 7.3 and 18.4 µM, respectively (Fig. 4A and Table II). The cytotoxicity EC_50_ values, representing mitochondrial toxicity, were 3.5, 3.4 and 11.8 µM, respectively (Table II).

The cell viability (%ATP amount) decreased in response to naphthoquinones and oligomycin in a concentration-dependent manner; this was accompanied by an increase in cytotoxicity (mitochondrial-derived dead cell protease) (Fig. 4A). However, these effects were almost abolished when cells were co-treated with the antioxidant NAC (Fig. 4B and Tables II and III, where Table II shows the results without NAC and Table III shows the results with NAC). These results revealed that naphthoquinone-induced cell death of drug-resistant cancer cells involved mitochondrial toxicity caused by ROS production. Mitochondrial toxicities that induce mitochondrial ROS and/or loss of transmembrane potential could cause pyroptosis (18), which is a process mediated by the pore-forming cleavage of gasdermin D. To further verify the properties of shikonin-induced cell death under OXPHOS, the presence of gasdermin D was analyzed. As a result, Shikonin induced the expression of gasdermin D under OXPHOS in R HSC-3 cells (Fig. 4C).

### Effects of necroptosis inhibitors on oxidative stress induced by naphthoquinones

**Figure 5.**
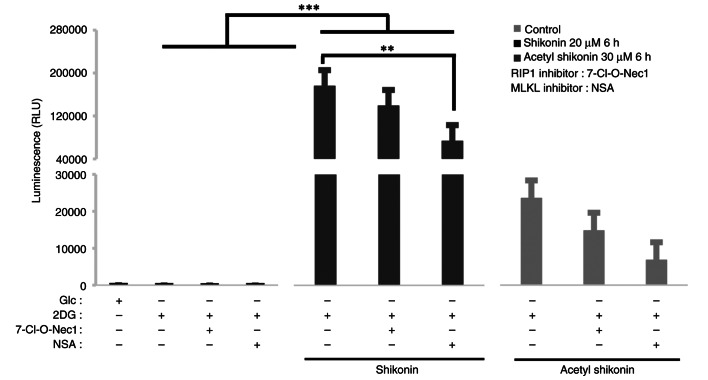
Effect of naphthoquinone-induced ROS production in combination with necroptosis inhibitors on R HSC-3 cells. ROS assay of R HSC-3 cells pretreated with RIP1 inhibitor (50 µM, 7-O-Cl-Nec1) and MLKL inhibitor (10 µM, necrosulfonamide) before naphthoquinone treatment (20 µM shikonin or 30 µM acetyl shikonin, 6 h) and cultured in OXPHOS conditions (culture medium containing 10 nM 2DG) for 24 h. Naphthoquinones (20 µM shikonin or 30 µM acetyl shikonin) were applied to necroptosis inhibitor pretreated R HSC-3 cells for 6 h, and ROS production was measured. Data are presented as the mean ± SD (n=3). **P<0.01, ***P<0.0005. ROS, reactive oxygen species; RIP1, receptor interacting protein 1 kinase; MLKL, mixed lineage kinase-domain like; OXPHOS, oxidative phosphorylation; 2DG, deoxy-D-glucose; Glc, glucose; NSA, necrosulfonamide; RLU, relative fluorescence units.

To explore the relationship between mitochondrial-derived ROS and necroptosis signals, 7-Cl-O-Nec1, a RIP1 inhibitor, and NSA, an MLKL inhibitor, were used as necroptosis inhibitors. RIP1 is a signaling molecule for early necroptosis, and MLKL is a kinase-like molecule that induces pore formation and destruction of the cell membrane (19–22). The ROS production induced by naphthoquinones was suppressed by pretreatment with the RIP1 and MLKL inhibitors (Fig. 5). Under 2DG culture conditions, shikonin treatment markedly increased ROS production. When compared with the 2DG + shikonin group, co-treatment with the RIP1 inhibitor slightly reduced ROS by 21% but this reduction was not statistically significant (P=0.661). By contrast, co-treatment with the MLKL inhibitor significantly reduced ROS levels by 59% compared with the 2DG + shikonin group (P=0.0019). For acetyl shikonin, ROS levels were reduced by 37% with RIP1 inhibitor (P=0.999) and 71% with MLKL inhibition (P=0.994) compared with the corresponding 2DG + acetyl shikonin group, but neither reduction reached statistical significance. These results suggest that ROS induction by naphtoquinones depends more strongly on MLKL than on RIP1, although the effect of acetyl shikonin was less pronounced.

### Induction of the damage-associated molecular pattern (DAMP) molecule calreticulin in R HSC-3 cells by shikonin

**Figure 6.**
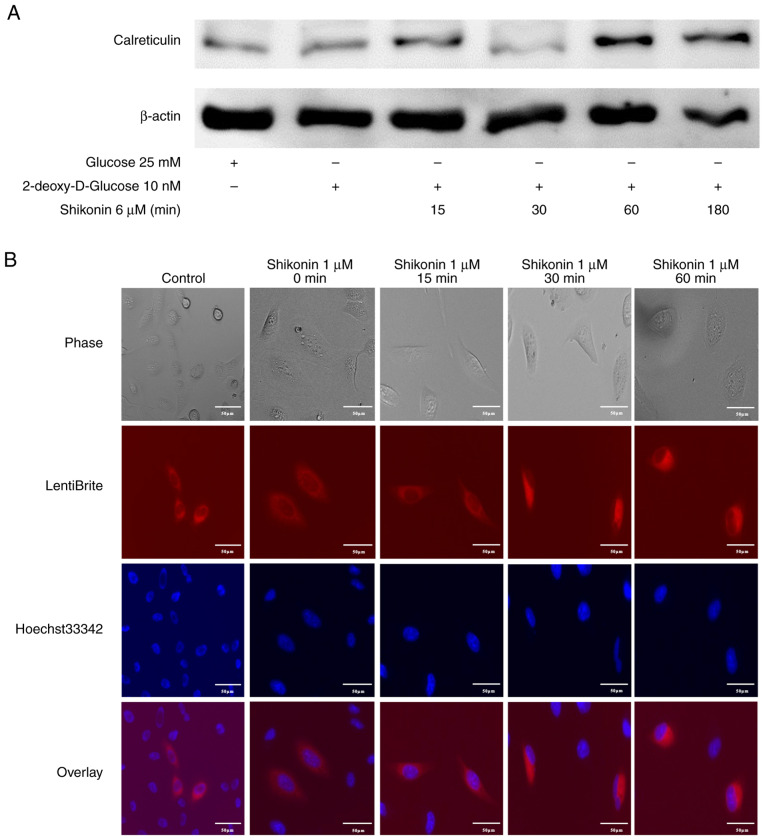
Expression of calreticulin in R HSC-3 cells treated with shikonin. (A) Western blot analysis of calreticulin was performed in R HSC-3 cells treated as indicated. (B) Fluorescence microscopy imaging of calreticulin in R HSC-3 cells infected with lentivirus expressing RFP-calreticulin at 15, 30 and 60 min after the addition of shikonin. Scale bar, 50 µm.

Cancer cells undergoing necroptotic cell death release DAMPs, which are immunogenic and potentiate cancer progression (23). DAMPs, such as extracellular release of ATP and exposure of calreticulin to the cell membrane surface, are important indicators of immunogenic cell death (ICD), a form of cell death that induces host immune responses. Calreticulin has been reported to act as an ‘eat me’ signal for dendritic cells (24,25). Therefore, it was examined whether necroptosis induced by naphthoquinones induces the expression of calreticulin, one of the ICD-related DAMP molecules. R HSC-3 cells cultured for 2 h in 2DG-DMEM, which induces OXPHOS, were treated with shikonin for 5 h and the expression of calreticulin was examined. The induction of calreticulin in R HSC-3 cells treated with shikonin was observed (Fig. 6A). Next, live cell imaging of intracellular calreticulin in R HSC-3 cells infected with lentivirus expressing calreticulin-RFP was performed. The fluorescence intensity in the cytoplasm increased after 30 min of shikonin treatment (Fig. 6B). This result indicated that drug-resistant cancer cell death caused by naphthoquinones may result in ICD, which induces tumor immunity.

## Discussion

In the present study, compounds that induce cell death in multidrug-resistant head and neck cancer cells were searched for. The investigation identified naphthoquinones and the mechanism of their cell death induction activity were further explored. These results may aid in the development of new therapeutic agents for multidrug-resistant head and neck cancer cells. In a cytostatic test using the R HSC-3 multidrug-resistant head and neck cancer cell line, naphthoquinones, including shikonin, were effective at reducing cell viability at a low concentration, with the IC_50_ values ≤3 times those of chemotherapeutic drugs. This suggests that naphthoquinones may be useful for head and neck cancer cells with acquired multidrug resistance. Shikonin, acetyl shikonin and β-hydroxyisovaleryl shikonin were analyzed; the latter two are naphthoquinones with different side chains. All tested naphthoquinones exhibited cytotoxic effects in cell viability assays. The IC_50_ results indicated that the side-chain OH group was related to the strength of cytotoxicity. All three naphthoquinones induced RIP1 expression in the R HSC-3 multidrug-resistant cancer cell line, suggesting that the common naphthoquinone skeleton was involved in the necroptosis-inducing activity.

The mechanism by which naphthoquinones induce necroptosis in multidrug-resistant head and neck cancer cells were further explored in the present study. Addition of the antioxidant, NAC, inhibited the cytotoxicity induced by naphthoquinones, suggesting that ROS, which cause oxidative stress, are involved in the cell death caused by naphthoquinones. In total, >90% of intracellular ROS are derived from mitochondria (11). Next, the mitochondrial membrane potential was measured using the membrane-permeable JC-1 dye. Treatment with naphthoquinones, except for shikonin, resulted in an accumulation of JC-1 monomers. These results suggest that naphthoquinones induce ROS production by altering the mitochondrial membrane potential. By contrast, another study reported that shikonin induces ROS production through mitochondrial depolarization (26). This suggests that the fluorescence of the mitochondrial membrane potential may be shorter than the measurement time, which may be why the changes in the mitochondrial membrane potential induced by shikonin were not detected in the present study. The absence of detectable mitochondrial membrane depolarization by shikonin may be due to transient changes that were not captured at the time of measurement or to the generation of ROS from non-mitochondrial sources such as NADPH oxidases (27,28).

Cancer cells predominantly generate ATP produced through glycolysis rather than mitochondrial OXPHOS, a phenomenon known as the Warburg effect (14). In the present study, to investigate the involvement of mitochondria in ROS induced by naphthoquinones, ROS were measured under conditions in which energy metabolism was tilted toward OXPHOS dominance. Naphthoquinones induced the generation of ROS in R HSC-3 cells in a concentration-dependent manner. ROS production was stronger with shikonin, showing a difference of >7-fold compared with acetyl shikonin. When the carbon source was changed from glycolysis-dependent glucose to mitochondrial-dominant galactose, treatment with naphthoquinones resulted in concentration-dependent decreases in ATP levels and increase in dead cell proteases. This suggests that cell death induced by naphthoquinones caused mitochondrial dysfunction manifested as a decrease in ATP levels. The increased mitochondrial toxicity and decreased ATP levels caused by naphthoquinones were recovered by the addition of NAC. This indicated that mitochondrial dysfunction caused by naphthoquinones was due to the production of ROS. The positive control oligomycin, an OXPHOS inhibitor (complex V inhibitor) was not affected by NAC. This suggests that naphthoquinones likely have an effect upstream of mitochondrial complex V to induce cell death.

To clarify the relationship between mitochondrial-derived ROS and necroptosis signals, two necroptosis inhibitors of RIP1 and MLKL were used. RIP1 is an early necroptosis signaling molecule, and MLKL is a kinase-like molecule that induces the formation and destruction of membrane pores (29). ROS production induced by naphthoquinones was suppressed by pretreatment with the necroptosis inhibitors. MLKL inhibition led to a higher inhibitory effect on ROS production than RIP1 inhibition. This suggests that naphthoquinone-induced ROS-induced cell death depends more on the MLKL execution factor than on RIP1. These results indicate that naphthoquinones induce necroptotic cell death in multidrug-resistant cancer cells by oxidative stress damage mediated by mitochondria-derived ROS.

**Figure 7.**
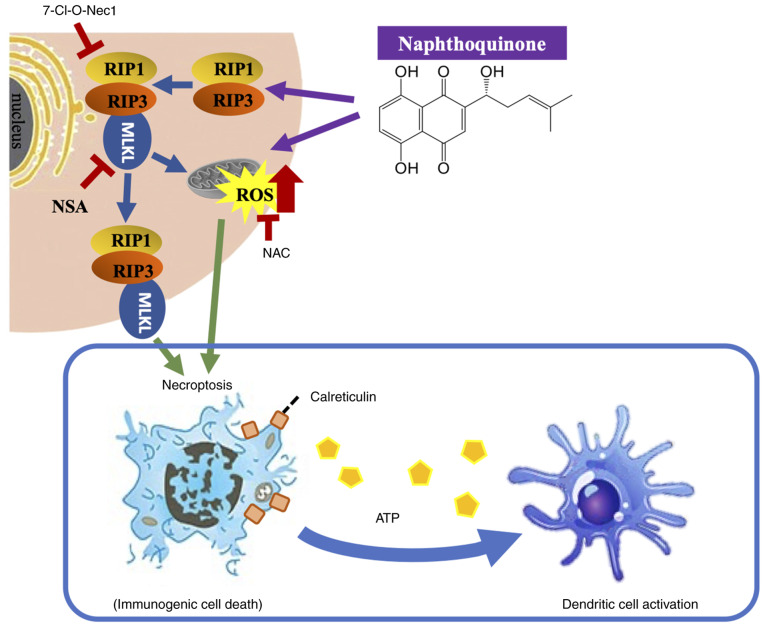
Induction of immunogenic cell death by naphthoquinones in R HSC-3 cells. Naphthoquinones cause cell death by direct or indirect mechanisms that induce mitochondrial dysfunction. RIP, receptor interacting protein; MLKL, mixed lineage kinase-domain like; NSA, necrosulfonamide; ROS, reactive oxygen species; NAC, *N*-acetyl cysteine.

The present study showed that naphthoquinones induce necroptosis in multidrug-resistant head and neck cancer cells. Furthermore, naphthoquinones induced necroptosis signals, including activation of the RIP1 and MLKL pathways, mediated by mitochondria-derived oxidative stress. The possibility of immunogenicity as a result of necroptosis was also suggested. In recent years, the induction of non-apoptotic cell death has attracted significant interest in the study of therapy-resistant and refractory cancer types. Among the cell death mechanisms, necroptosis has been increasingly recognized as a distinct form of programmed cell death (30,31). RIP1, RIP3 and MLKL have been identified as regulators of necroptosis (32–35). A signal transduction pathway through the RIP1-RIP3-MLKL complex plays a key role in necroptosis (36–41). Necroptosis causes cell rupture as a result of morphological changes such as cell swelling, mitochondrial swelling or rupture and organelle membrane disruption, and subsequently cell death with the release of DAMPs (35,42–45). In cancer cells, DAMPs act on immune cells and are thought to strengthen their ability to attack cancer (23). ICD is a type of programmed cell death that stimulates an antitumor immune response. Necroptosis has emerged as a key contributor to ICD due to its ability to induce the release of immunostimulatory signals. DAMPs, such as the exposure of calreticulin to the cell membrane surface and the extracellular release of ATP, are important indicators of ICD (46). Calreticulin acts primarily as an ‘eat me’ signal to dendritic cells (24,25), and cancer-specific antigens are presented to cytotoxic T cells via dendritic cells, contributing to the activation of immune cells with high attack power against cancer (25,47). A previous transcriptomic analysis showed that patients with head and neck squamous cell carcinoma with high ICD-related gene signatures have an improved prognosis and responsiveness to immune checkpoint inhibitors compared with those with low ICD-related gene signatures (48) This supports the potential clinical value of ICD-inducing agents such as shikonin in combination with immunotherapy. In the present study, it was found that shikonin induced necroptosis in multidrug-resistant head and neck cancer cells and induced the expression of calreticulin in a concentration-dependent manner (Fig. 7).

The present study has several limitations. First, whether NAC affects the uptake or metabolism of napthoquinones was not assessed, which could potentially influence the interpretation of its ROS-scavenging effects. Additionally, NAC is primarily recognized as a ROS scavenger, and its influence on shikonin pharmacokinetics is considered minimal but cannot be completely ruled out (49,50). In the present study, it was also observed that shikonin induced ROS production but did not alter the mitochondrial membrane potential at 2 h post-treatment, suggesting that any membrane depolarization might have been transient or undetectable at this time point. Moreover, only *in vitro* experiments were conducted in the present study. Future studies using animal models, such as immunodeficient mice bearing xenografts of drug-resistant head and neck squamous cell carcinoma cells, are required to validate the antitumor activity of naphthoquinones in this disease. Further research should also examine whether naphthoquinones influence tumor immunity.

In conclusion, the present study demonstrated that mitochondrial-derived ROS-mediated oxidative stress damage by naphthoquinones caused necroptotic cell death in multidrug-resistant head and neck cancer cells. Given the high levels of ROS induced by naphthoquinones, the potential for systemic toxicity *in vivo* must be carefully evaluated. Dose-escalation studies and histopathological analysis of major organs (such as liver, kidney and heart) should be performed to assess safety. Co-administration of antioxidants such as NAC may help mitigate off-target oxidative damage without compromising antitumor efficacy (49,50). Necroptotic cell death by shikonin releases DAMPs from multidrug-resistant cancer cells, and these may contribute to immunogenicity (Fig. 7). This suggests that the ICD of metastatic oral cancer with acquired multidrug resistance, which is an intractable cancer, may be a target of the immune system and induce cytotoxic T cells with higher selectivity. The effect of naphthoquinones on improving the response rate of head and neck cancer to immunotherapy should be explored in future studies.

## Figures and Tables

**Table I.** IC_50_ values in drug-resistant and parental HSC-3 cells.

Drug	Unit	IC_50_ in HSC-3^a^ (µM)	IC_50_ in R HSC-3^a^ (µM)	P-value
Cisplatin	µM	0.68±0.17	20.30±6.32	P<0.001
SN-38	nM	7.11±1.68	22.18±3.70	P<0.001
Docetaxel	nM	0.90±0.20	6.57±1.16	P=0.002
Erlotinib	µM	0.21±0.04	31.90±2.00	P<0.001
Doxorubicin	nM	26.57±6.00	223.63±15.70	P=0.028
Shikonin	µM	4.37±0.53	8.10±0.58	P=0.003
Acetyl shikonin	µM	7.83±1.40	23.90±6.50	P<0.001

a Values are presented as the mean ± SD (n≥3). R HSC-3, drug-resistant HSC-3 cells; IC_50_, half maximal inhibitory concentration.

**Table II.** Effects of naphthoquinones under mitochondrial oxidative phosphorylation conditions was evaluated by Mitochondrial ToxGlo^™^ assay.

Drug	Cytotoxicity EC_50_ (µM)	ATP EC_50_ (µM)
Shikonin	3.47	7.18
Acetyl shikonin	3.43	7.27
β-Hydroxy isovaleryl shikonin	11.77	18.43
Oligomycin^a^	6.45	3.44

a Oligomycin was the positive control. EC_50_, half maximal effective concentration.

**Table III.** Effects of naphthoquinones under mitochondrial oxidative phosphorylation conditions and in the presence of the antioxidant, *N*-acetyl cysteine, was evaluated by Mitochondrial ToxGlo^™^ assay.

Drug	Cytotoxicity EC_50_ (µM)	ATP EC_50_ (µM)
Shikonin	>10.00	>10.00
Acetyl shikonin	>20.00	>10.00
β-Hydroxy isovaleryl shikonin	>50.00	>50.00
Oligomycin^a^	5.57	2.42

a Oligomycin was the positive control. EC_50_, half maximal effective concentration.

## Data Availability

The data generated in the present study may be requested from the corresponding author.

## Authors' information

Naoki Umemura, http://orcid.org/0000-0002-3249-782X; Hiromi Miyazaki, https://orcid.org/0000-0001-7719-4913; Makoto Adachi, http://orcid.org/0000-0002-9382-7052; Emika Ohkoshi, http://orcid.org/0000-0001-9700-9973.

## References

[1] Sung H, Ferlay J, Siegel RL, Laversanne M, Soerjomataram I, Jemal A, Bray F (2021). Global cancer statistics 2020: GLOBOCAN estimates of incidence and mortality worldwide for 36 cancers in 185 countries. CA Cancer J Clin.

[2] Argiris A, Karamouzis MV, Raben D, Ferris RL (2008). Head and neck cancer. Lancet.

[3] Price KA, Cohen EE (2012). Current treatment options for metastatic head and neck cancer. Curr Treat Options Oncol.

[4] Chang JH, Wu CC, Yuan KS, Wu ATH, Wu SY (2017). Locoregionally recurrent head and neck squamous cell carcinoma: Incidence, survival, prognostic factors, and treatment outcomes. Oncotarget.

[5] Sugimoto Y, Tsukahara S, Sato S, Suzuki M, Nunoi H, Malech HL, Gottesman MM, Tsuruo T (2003). Drug-selected co-expression of P-glycoprotein and gp91 in vivo from an MDR1-bicistronic retrovirus vector Ha-MDR-IRES-gp91. J Gene Med.

[6] Murakami K, Umemura N, Adachi M, Motoki M, Ohkoshi E (2022). ABCG2, CD44 and SOX9 are increased with the acquisition of drug resistance and involved in cancer stem cell activities in head and neck squamous cell carcinoma cells. Exp Ther Med.

[7] Smith ER, Wang JQ, Yang DH, Xu XX (2022). Paclitaxel resistance related to nuclear envelope structural sturdiness. Drug Resist Updat.

[8] Kanda Y (2013). Investigation of the freely available easy-to-use software ‘EZR’ for medical statistics. Bone Marrow Transplant.

[9] Ofengeim D, Yuan J (2013). Regulation of RIP1 kinase signalling at the crossroads of inflammation and cell death. Nat Rev Mol Cell Biol.

[10] Xie Y, Zhao G, Lei X, Cui N, Wang H (2023). Advances in the regulatory mechanisms of mTOR in necroptosis. Front Immunol.

[11] Murphy MP (2009). How mitochondria produce reactive oxygen species. Biochem J.

[12] Butow RA, Avadhani NG (2004). Mitochondrial signaling: The retrograde response. Mol Cell.

[13] Sena LA, Chandel NS (2012). Physiological roles of mitochondrial reactive oxygen species. Mol Cell.

[14] Warburg O (1956). On the origin of cancer cells. Science.

[15] Pfeiffer T, Schuster S, Bonhoeffer S (2001). Cooperation and competition in the evolution of ATP-producing pathways. Science.

[16] Marroquin LD, Hynes J, Dykens JA, Jamieson JD, Will Y (2007). Circumventing the Crabtree effect: Replacing media glucose with galactose increases susceptibility of HepG2 cells to mitochondrial toxicants. Toxicol Sci.

[17] Niles AL, Moravec RA, Eric Hesselberth P, Scurria MA, Daily WJ, Riss TL (2007). A homogeneous assay to measure live and dead cells in the same sample by detecting different protease markers. Anal Biochem.

[18] Miao R, Jiang C, Chang WY, Zhang H, An J, Ho F, Chen P, Zhang H, Junqueira C, Amgalan D (2023). Gasdermin D permeabilization of mitochondrial inner and outer membranes accelerates and enhances pyroptosis. Immunity.

[19] Rickard JA, O'Donnell JA, Evans JM, Lalaoui N, Poh AR, Rogers T, Vince JE, Lawlor KE, Ninnis RL, Anderton H (2014). RIPK1 regulates RIPK3-MLKL-driven systemic inflammation and emergency hematopoiesis. Cell.

[20] Dillon CP, Weinlich R, Rodriguez DA, Cripps JG, Quarato G, Gurung P, Verbist KC, Brewer TL, Llambi F, Gong YN (2014). RIPK1 blocks early postnatal lethality mediated by caspase-8 and RIPK3. Cell.

[21] Huang Z, Zhou T, Sun X, Zheng Y, Cheng B, Li M, Liu X, He C (2018). Necroptosis in microglia contributes to neuroinflammation and retinal degeneration through TLR4 activation. Cell Death Differ.

[22] Yu Z, Jiang N, Su W, Zhuo Y (2021). Necroptosis: A novel pathway in neuroinflammation. Front Pharmacol.

[23] Wellenstein MD, de Visser KE (2018). Cancer-cell-intrinsic mechanisms shaping the tumor immune landscape. Immunity.

[24] Gardai SJ, McPhillips KA, Frasch SC, Janssen WJ, Starefeldt A, Murphy-Ullrich JE, Bratton DL, Oldenborg PA, Michalak M, Henson PM (2005). Cell-surface calreticulin initiates clearance of viable or apoptotic cells through trans-activation of LRP on the phagocyte. Cell.

[25] Tesniere A, Apetoh L, Ghiringhelli F, Joza N, Panaretakis T, Kepp O, Schlemmer F, Zitvogel L, Kroemer G (2008). Immunogenic cancer cell death: A key-lock paradigm. Curr Opin Immunol.

[26] Lee MJ, Kao SH, Hunag JE, Sheu GT, Yeh CW, Hseu YC, Wang CJ, Hsu LS (2014). Shikonin time-dependently induced necrosis or apoptosis in gastric cancer cells via generation of reactive oxygen species. Chem Biol Interact.

[27] Forrester SJ, Kikuchi DS, Hernandes MS, Xu Q, Griendling KK (2018). Reactive oxygen species in metabolic and inflammatory signaling. Circ Res.

[28] Li J, Pang J, Liu Z, Ge X, Zhen Y, Jiang CC, Liu Y, Huo Q, Sun Y, Liu H (2021). Shikonin induces programmed death of fibroblast synovial cells in rheumatoid arthritis by inhibiting energy pathways. Sci Rep.

[29] Wu XN, Yang ZH, Wang XK, Zhang Y, Wan H, Song Y, Chen X, Shao J, Han J (2014). Distinct roles of RIP1-RIP3 hetero- and RIP3-RIP3 homo-interaction in mediating necroptosis. Cell Death Differ.

[30] Christofferson DE, Yuan J (2010). Necroptosis as an alternative form of programmed cell death. Curr Opin Cell Biol.

[31] Smith CC, Yellon DM (2011). Necroptosis, necrostatins and tissue injury. J Cell Mol Med.

[32] Degterev A, Hitomi J, Germscheid M, Ch'en IL, Korkina O, Teng X, Abbott D, Cuny GD, Yuan C, Wagner G (2008). Identification of RIP1 kinase as a specific cellular target of necrostatins. Nat Chem Biol.

[33] Cho YS, Challa S, Moquin D, Genga R, Ray TD, Guildford M, Chan FK (2009). Phosphorylation-driven assembly of the RIP1-RIP3 complex regulates programmed necrosis and virus-induced inflammation. Cell.

[34] Sun L, Wang H, Wang Z, He S, Chen S, Liao D, Wang L, Yan J, Liu W, Lei X, Wang X (2012). Mixed lineage kinase domain-like protein mediates necrosis signaling downstream of RIP3 kinase. Cell.

[35] Linkermann A, Green DR (2014). Necroptosis. N Engl J Med.

[36] Zhao J, Jitkaew S, Cai Z, Choksi S, Li Q, Luo J, Liu ZG (2012). Mixed lineage kinase domain-like is a key receptor interacting protein 3 downstream component of TNF-induced necrosis. Proc Natl Acad Sci USA.

[37] Wang H, Sun L, Su L, Rizo J, Liu L, Wang LF, Wang FS, Wang X (2014). Mixed lineage kinase domain-like protein MLKL causes necrotic membrane disruption upon phosphorylation by RIP3. Mol Cell.

[38] Moreno-Gonzalez G, Vandenabeele P, Krysko DV (2016). Necroptosis: A novel cell death modality and its potential relevance for critical care medicine. Am J Respir Crit Care Med.

[39] Quarato G, Guy CS, Grace CR, Llambi F, Nourse A, Rodriguez DA, Wakefield R, Frase S, Moldoveanu T, Green DR (2016). Sequential engagement of distinct MLKL Phosphatidylinositol-binding sites executes necroptosis. Mol Cell.

[40] Dhuriya YK, Sharma D (2018). Necroptosis: A regulated inflammatory mode of cell death. J Neuroinflammation.

[41] Green DR (2019). The coming decade of cell death research: Five riddles. Cell.

[42] Aaes TL, Kaczmarek A, Delvaeye T, De Craene B, De Koker S, Heyndrickx L, Delrue I, Taminau J, Wiernicki B, De Groote P (2016). Vaccination with necroptotic cancer cells induces efficient anti-tumor immunity. Cell Rep.

[43] Gong YN, Guy C, Crawford JC, Green DR (2017). Biological events and molecular signaling following MLKL activation during necroptosis. Cell Cycle.

[44] Grootjans S, Vanden Berghe T, Vandenabeele P (2017). Initiation and execution mechanisms of necroptosis: An overview. Cell Death Differ.

[45] Galluzzi L, Vitale I, Aaronson SA, Abrams JM, Adam D, Agostinis P, Alnemri ES, Altucci L, Amelio I, Andrews DW (2018). Molecular mechanisms of cell death: Recommendations of the nomenclature committee on cell death 2018. Cell Death Differ.

[46] Zhang Y, Liu L, Jin L, Yi X, Dang E, Yang Y, Li C, Gao T (2014). Oxidative stress-induced calreticulin expression and translocation: New insights into the destruction of melanocytes. J Invest Dermatol.

[47] Obeid M, Tesniere A, Ghiringhelli F, Fimia GM, Apetoh L, Perfettini JL, Castedo M, Mignot G, Panaretakis T, Casares N (2007). Calreticulin exposure dictates the immunogenicity of cancer cell death. Nat Med.

[48] Wang X, Wu S, Liu F, Ke D, Wang X, Pan D, Xu W, Zhou L, He W (2021). An Immunogenic cell death-related classification predicts prognosis and response to immunotherapy in head and neck squamous cell carcinoma. Front Immunol.

[49] Bauza-Thorbrugge M, Peris E, Zamani S, Micallef P, Paul A, Bartesaghi S, Benrick A, Wernstedt Asterholm I (2023). NRF2 is essential for adaptative browning of white adipocytes. Redox Biol.

[50] Chen S, Ren Q, Zhang J, Ye Y, Zhang Z, Xu Y, Guo M, Ji H, Xu C, Gu C (2014). N-acetyl-L-cysteine protects against cadmium-induced neuronal apoptosis by inhibiting ROS-dependent activation of Akt/mTOR pathway in mouse brain. Neuropathol Appl Neurobiol.

